# A study revealing volatile aroma produced by *Pediococcus pentosaceus* in dough fermentation

**DOI:** 10.1002/fsn3.1807

**Published:** 2020-08-03

**Authors:** Yunxia Gong, Xiaobao Qi

**Affiliations:** ^1^ College of Food Science and Technology Huazhong Agricultural University Wuhan China

**Keywords:** extracellular proteins, microbial interaction, *P. pentosaceus*, protease activity, volatile aroma

## Abstract

*Pediococcus pentosaceus* is important probiotics in Chinese Laomian. Its role in meat and fermented vegetable has been largely demonstrated, but few studies have investigated the role of *P. pentosaceus* in Chinese Laomian. For this purpose, we simulated Laomian fermentation using *Saccharomyces cerevisiae* and *P. pentosaceus*. Volatile aroma was detected by headspace solid‐phase microextraction gas–chromatography‐mass spectrometry. Real‐time fluorescent quantitative polymerase chain reaction was used to determine dynamic growth of *S. cerevisiae* and *P. pentosaceus* in fermentation. Extracellular proteins were analyzed by sodium dodecyl sulfate–polyacrylamide gel electrophoresis. Folin–Ciocalteu method was used to detect extracellular protease activity in different pH values. Owing to addition of *P. pentosaceus,* the types and contents of esters increase, the relative contents of acetic acid hexyl ester, formic acid octyl ester, and heptanoic acid ethyl ester rise obliviously; especially, the relative content of hexanoic acid ethyl ester was highly correlated with *P. pentosaceus* by increasing 20.61%. As the gel electrophoresis results display, due to mixed fermentation of *S. cerevisiae* and *P. pentosaceus*, the 25k Da and 51k Da proteins expression quantity of *P. pentosaceus* clearly increased. Under neutral and alkaline culture conditions, the extracellular protease activity of *P. pentosaceus* is higher. This research benefits to gain insight into the fermentation actions of *P. pentosaceus* in Chinese Laomian.

## Graphical abstract

This research systematically explores the fermentation characteristics of *P. pentosaceus* in Chinese traditional Laomian, the interaction between *S. cerevisiae* and *P. pentosaceus*, gain insight of the fermentation characteristics of *P. pentosaceus* and benefits the development of Chinese traditional Laomian bread.

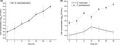

## 1 INTRODUCTION

Flavor is the first consideration for consumer (Salim‐ur‐Rehman, Paterson, & Piggott, 2006), Chinese traditional bread has been popular for more than 1,000 years mainly due to unique flavor (Ding, 2007). Previous research shows that yeast fermentation was to contribute to sponge texture (Zhang, Sun, Sadiq, Sakandar, & He, 2018). Lactic acid bacteria (LAB) were to improve aroma, nutritional value, and shelf life of bread (Michalska et al., 2007). There are many kinds of LAB in the Chinese Laomian (sourdough) such as *Lactobacillus plantarum, Leuconostoc mesenteroides, and P. pentosaceus*. (Zhang, Liu, Liu, Luo, & Xiao‐Ran, 2016). Our study also finds that there is relatively high ratio for *P. pentosaceus* (*P. pentosaceus)* in the Chinese Laomian (*P. pentosaceus* was detected in eight samples from 13 Chinese traditional Laomian). *Pediococcus pentosaceus* were commonly found in dairy products (Yu, Chen, Tao, Bo, & Mu, 2015), meat products (Anastasiadou, Papagianni, Filiousis, Ambrosiadis, & Koidis, 2008), and vegetable fermentation products (Xu, Wenshui, Fang, & Xiaohua, 2010), and mainly helpful to the quality, safety, flavor, and shelf life of fermented products (Kwon, Jo, & Park, 2018). But few studies reported action of *P. pentosaceus* in the dough fermentation. In this study, we systematically studied the action mechanism of *P. pentosaceus* in the fermentation dough by adding *S. cerevisiae* (*S. cerevisiae)* alone or *S. cerevisiae* and *P. pentosaceus* joint as a starter to simulate the dough fermentation. Headspace solid‐phase microextraction–gas chromatography‐mass spectrometry (HS‐SPME‐GC‐MS) was used as a detection method of volatile aroma substances to analyze the contribution of *P. pentosaceus* to the fermentation aroma. Real‐time fluorescent quantitative polymerase chain reaction (RT‐qPCR) was used to detect the dynamic changes of *S. cerevisiae* and *P. pentosaceus* during the simulated fermentation process to explore their interaction. Sodium dodecyl sulfate–polyacrylamide gel electrophoresis (SDS‐PAGE) protein gel electrophoresis was used to analyze extracellular proteins, and Folin–Ciocalteu method was used to detect extracellular protease activity. Therefore, this study benefits to explore the interaction between *S. cerevisiae* and *P. pentosaceus*, and gain insight of the fermentation characteristics of *P. pentosaceus*.

## 2 MATERIALS AND METHODS

### 2.1 Materials

*Saccharomyces cerevisiae* and *P. pentosaceus* were obtained from our laboratory isolated from Chinese Laomian (sourdoughs), their cultures were maintained and revived as described by *Handbook of Culture Media for Food Microbiology* ("de man, rogosa, and sharpe (MRS) agar," 2003)*. Saccharomyces cerevisiae* and *P. pentosaceus* were cultured to mid‐log growth phase by yeast extract peptone dextrose (YPD) and de Man, Rogosa and Sharpe (MRS) liquid media, the cells were collected by centrifuge, washed twice with normal saline, resuspended in normal saline, and respectively adjusted by spectrophotometry to 1.00/0.40 (OD_600nm_), and final cell quantities were confirmed by plate culture.

Flour was purchased from Wonder Farm; PC0030 lowry protein concentration determination kit was purchased from Solarbio; A Supelco fiber (Carboxen/DVB/PDMS 50/30 μm) and SPME were purchased from Supelco; The 6890N‐5975 GC‐MS instrument and micro‐sampler (10 μl) were purchased from Agilent Corporation; Cooling Column Thermostat 0.2 μm cellulose acetate membrane was purchased from Sartorius Stedim; and the other general chemical reagents were purchased from China National Pharmaceutical Group Corporation. The specific primers sequences of *S. cerevisiae* and *P. pentosaceus* are respectively referred to CESP‐F/SCERR (Hierro, Esteve‐Zarzoso, Gonzalez, Mas, & Guillamon, 2006) and PedPen3 (Stevenson, Muck, Shinners, & Weimer, 2006). Primers were synthesized by Sangong Biotech Company.

### 2.2 Simulated dough fermentation

To simulate fermentation process of the Chinese traditional Laomian, we prepared 10% (*w/v*) sterile flour solution (SFS) as flour fermented system, 10% (*w/v*) SFS preparation are following: 100 g of flour added into 1,000 ml distilled water, stirred evenly and sterilized at 121°C for 20 min. The simulated fermentation group consisted of 20 ml of SFS and 1 ml strains solution including *S. cerevisiae* alone or *S. cerevisiae* and *P. pentosaceus* joint and then incubated to double start volume. Each test was repeated three times independently, and each time has three repeats.

### 2.3 Determination of aroma in simulated fermentation group

Volatile aroma of fermented dough was detected by HS‐SPME‐GC‐MS. Firstly, a Supelco fiber was conditioned according to the manufacturer's instructions. Secondly, after equilibrating at 40°C for 20 min, the fiber was exposed to 5.00 g of simulated fermentation group and 5 ml sterile water in a 20‐ml glass vial containing a micro‐stirring bar for 40‐min extraction. In the end, after volatiles were extracted, the fiber was inserted into the gas chromatography (GC) injector at 250°C to desorb the analytes for 5 min (Qiao et al., 2012). Volatile aroma substances were subjected to GC analysis coupled to a mass spectrometer. The mass spectrometer was operated in an electron ionization mode at a voltage of 70 eV. The flow rate of helium on the HP‐5MS column was 1.0 ml/min. Analysis was conducted in a splitless mode. The column was initially maintained at 40°C for 3 min; temperature was then increased from 40°C to 160°C at 3°C/min speed and finally increased to 220°C at a rate of 8°C/min. Temperature was maintained at 220°C for 3 min. The retention index of each chromatographic peak was calculated using the retention time of the C6‐C25 n‐alkanes. The analysis was performed by using computer spectra (NIST05/WILE7.0) for preliminary search and data analysis, combined with the retention index of the literature for comparison and manual spectrometer analysis to confirm the composition of volatile substances.

### 2.4 The population dynamic change of *S. cerevisiae* and P. pentosaceus during fermentation

The cell dynamic concentrations were detected by RT‐qPCR to explore the interaction between *S. cerevisiae* and *P. pentosaceus* during the simulated fermentation process. RT‐qPCR and standard curves were carried out using the previous count methods with some small modification (Pontonio et al., 2017). The standard curves of *S. cerevisiae* and *P. pentosaceus* were constructed using the quantification cycle (Ct) versus different cell numbers of strains. During fermentation progress, each 4 hr, 10 g of simulated fermentation dough was removed into 90 ml of sterile distilled water, followed by mixing and centrifuging at 300 × *g* for 10 min, 1 ml of supernatant was transferred to a new tube and centrifuged for 10 min (4°C, 10,000 × *g*), the pellets were collected and washed twice by sterile water for DNA extraction. Each strain concentration was calculated by RT‐qPCR assay, each test was repeated three times independently, and each time has three repeats.

### 2.5 Extracellular protein composition of *S. cerevisiae* and *P. pentosaceus* by SDS‐PAGE

To investigate the roles of extracellular protein during dough fermentation, deoxycholate–trichloroacetic acid（TCA‐DOC）method was used to extract extracellular proteins with some light improvement (Wang, He, Huang, & Qingmei, 2014). In brief, the strains were cultured in 400 ml MRS broth at 5% inoculum (*w/w*) overnight at 37°C, centrifuged (3,000 × *g*, 10 min) to collect the supernatant, and 40 ml 2% (*w/w*) DOC solution was added and kept for 30 min at 4°C. TCA was added to form final 6% TCA solution, and the solution was allowed to stand at 4°C for 4 hr and centrifuged (4°C, 10,000 × *g*, 15 min) to collect the precipitate. The precipitate was ultrasonically washed three times with 10 ml ice acetone (stored at −20°C), naturally air‐dried at room temperature in the end. All samples were adjusted to a uniform total concentration by quick start bradford protein assay kit (Bio‐Rad) using bovine serum albumin (BSA) as a standard. Sodium dodecyl sulfate–polyacrylamide gel electrophoresis (SDS‐PAGE) was carried using 12% resolving gel and 5% stacking gel. Standard molecular weight makers (Solarbio) were used to estimate the molecular weight of the extracellular protein (Yanshun, Dai, Zang, Jiang, & Xia, 2015). Each test was repeated three times independently, and each time has three repeats.

### 2.6 Extractcellular protease activity of *P. pentosaceus*

The extracellular protease activity was quantified under different pH conditions as described by Bhaskar et al. with minor modification (Bhaskar, Sudeepa, Rashmi, & Tamil Selvi, 2007). In brief, protease activity of *P. pentosaceus* was determined using casein as substrate at 40°C according to the instructions of lowry protein concentration determination kit. One unit of proteolytic activity (U) was defined as microgram tyrosine liberated per min of the enzyme extract. Specific activity was expressed as units per miligram protein (U/mg protein) of the enzyme extract. The standard curve of extracellular protease activity was generated by plotting the absorbance (OD_680nm_) versus different concentrations of casein (μg/mL). The *P. pentosaceus* cells were washed twice with normal saline and adjusted to 10^9^ CFU/mL as inoculum. 500 μl of *P. pentosaceus* was respectively inoculated into 10 ml MRS medium in pH 5, 6, 7 and 8) and incubated at 37°C for 24 hr, followed by centrifuging at 10,000 × *g* for 15 min to separate the biomass from supernatant. The supernatant was then collected and filtered through a 0.2‐μm cellulose acetate membrane to obtain cell‐free supernatant (CFS), which was used as extracellular enzyme for the determination of protease activity. Each test was repeated three times independently, and each time has three repeats.

### 2.7 Statistical analysis

Microbiological data were log transformed before statistical treatment. The data were expressed as the means ± standard error of biological triplicates. A completely randomized design was used to plan the flavor study.

## 3 RESULTS AND DISCUSSION

### 3.1 Aroma analysis produced by simulated fermentation groups

In order to detect the contribution of *P. pentosaceus* to aroma substances in the fermented dough, we simulated fermentation dough by *S. cerevisiae* alone or *S. cerevisiae* and *P. pentosaceus* joint as starter, and HS‐SPME‐GC‐MS was performed to detect the aroma substances. The (Figure S1) showed the 2‐pentylfuran, a fruit‐aroma substance (Kati, Kaisa, & Karin, 2007), was detected at extremely high level in *S. cerevisiae* alone dough, the content of hexanoic acid ethyl ester and 1‐pentanol increased obviously in *S. cerevisiae* and *P. pentosaceus* joint fermentation dough. The detailed results were shown in Table 1, there were total 19 kinds of aroma substances detected in *S. cerevisiae* alone fermented dough, and 34 volatile aroma substances in jointly fermented dough. Firstly, alcohols include 1‐pentanol, 1‐hexanol, 1‐octen‐3‐ol, 1‐octanol and phenylethyl alcohol, which appear in the both fermented doughs. However, the relative content of 1‐pentanol obviously increased and the others lessen due to the addition of *P. pentosaceus*. Secondly, three kinds of organic acids were also detected in both, and the relative contents of volatile organic acids decreased slightly because of the addition of *P. pentosaceus*. Thirdly, the main differences of volatile aroma were the contents and types of esters, hydrocarbons, and other substances. In *S. cerevisiae* alone fermentation dough, only six kinds of esters were detected and their contents are relatively low, with the addition of *P. pentosaceus*, additional eight kinds of esters were detected. To our surprise, the relative content of hexanoic acid ethyl ester dramatically increases from 9.12% to 29.73%, while 2‐pentylfuran significantly decreases from 26.71% to 2.8%. Generally, due to the addition of *P. pentosaceus*, the contents and types of volatile compounds esters increased and organic acids contents weakly declined. 2‐pentylfuran is a characteristic aroma substance of *S. cerevisiae*, and hexanoic acid ethyl ester is a characteristic aroma of *P. pentosaceus*.

**TABLE 1:** The aroma compounds detected by HS‐SPME‐GC‐MS

RT (min)	Substance	*S. cerevisiae* alone (Content %)	*S. cerevisiae and P. pentosaceus* (Content %)
	Alcohol		
5.416	1‐Pentanol	1.21 ± 0.02	9.22 ± 0.12
11.164	1‐Hexanol	7.89 ± 0.079	1.6 ± 0.031
11.222	1‐Octen−3‐ol	3.21 ± 0.072	0.51 ± 0.0064
11.594	1‐Octanol	2.21 ± 0.081	1.45 ± 0.012
15.896	Phenylethyl alcohol	7.6 ± 0.051	4.12 ± 0.011
	Esters		
2.021	Acetic acid ethyl ester	3.91 ± 0.030	1.19 ± 0.046
6.575	Formic acid, hexyl ester	0.1 ± 0.010	0.48 ± 0.0083
8.183	Pentanoic acid, ethyl ester	0.09 ± 0.0061	0.6 ± 0.0060
12.212	Hexanoic acid, ethyl ester	9.12 ± 0.035	29.73 ± 0.16
13.196	Acetic acid, hexyl ester	‐	0.61 ± 0.0025
15.959	Formic acid, octyl ester	‐	1.17 ± 0.015
17.258	Heptanoic acid, ethyl ester	‐	2.93 ± 0.067
21.91	Octanoic acid, ethyl ester	‐	5.52 ± 0.057
21.979	Undecanoic acid, ethyl ester	‐	3.08 ± 0.016
23.512	Butanedioic acid, dimethyl ester	‐	0.16 ± 0.0018
24.188	2‐Octenoic acid, ethyl ester	‐	0.09 ± 0.0020
24.296	Hexanoic acid, 3‐methylbutyl ester	‐	0.2 ± 0.0015
26.522	Nonanoic acid, ethyl ester	0.33 ± 0.020	2.1 ± 0.027
30.779	Decanoic acid, ethyl ester	0.21 ± 0.015	0.7 ± 0.0017
	Organic acid		
3.548	Acrylic acid	2.15 ± 0.020	1.12 ± 0.015
6.993	Oxalic acid	2.11 ± 0.025	0.79 ± 0.0032
13.222	Octanoic acid	3.98 ± 0.023	2.71 ± 0.016
	Hydrocarbons		
4.664	Octane	0.2 ± 0.015	0.73 ± 0.0064
6.381	Cyclohexane	0.1 ± 0.011	0.25 ± 0.0052
6.982	Cyclopropane, propyl‐	‐	3.49 ± 0.015
7.182	1‐Hexene, 2‐methyl‐	1.1 ± 0.14	4.5 ± 0.031
13.762	2‐Octene, 2,6‐dimethyl‐	‐	0.82 ± 0.0046
15.17	1‐Octyne	‐	0.26 ± 0.0025
24.668	Bicyclo[2.2.1]heptane, 2‐methyl‐exo‐	‐	0.12 ± 0.0016
	Others		
11.88	3‐Octanone	0.09 ± 0.0050	0.24 ± 0.0052
12.017	Furan, 2‐pentyl	26.71 ± 0.44	2.8 ± 0.056
24.949	1H‐Inden−1‐one, octahydro−7a‐hydroxy‐	‐	0.29 ± 0.0066
26.236	2‐Cyclohexen−1‐one, 4,4,6‐trimethyl‐	‐	0.14 ± 0.0015
26.31	6‐Octen−1‐ol, 3,7‐dimethyl‐, 1‐formate	‐	0.23 ± 0.0051

Note

Comparison of partial aroma components produced by simulated fermentation groups of dough with *S. cerevisiae* alone or *S. cerevisiae* and *P. pentosaceus* joint. RT means the retention time of each aroma component by HS‐SPME‐GC‐MS analysis. The dash (‐) represents the specific aroma compound was not detected.

Wheat flour is rich in nutrients and provides a rich source of carbon and nitrogen for microorganisms, one key driver of aroma is the carbon source available to the microorganisms, the low‐molecular weight sugars is, at least in part, aroma precursors (Samoticha, Wojdyło, Chmielewska, & Nofer, 2019). *Saccharomyces cerevisiae* can convert carbohydrates, lipids, and amino acids into volatile aroma substances includes alcohol, esters, organic acids, hydrocarbons, and some other compounds (e.g., 3‐octanone and 2‐pentylfuran) during the growth and metabolism process, which constitute the basic volatile aroma substances in dough (De Vuyst et al., 2014). *Pediococcus pentosaceus* (facultatively heterofermentative) metabolize hexose through EMP pathway and produce organic acids, metabolize pentose through HMP pathway, generate ethanol and acetic acid, and degrade leucine and phenylalanine to produce alcohols. Esters are derived from the esterification between acids and alcohols, for example, acetic acid, propionic acid, and butyric acid are usually formed with different alcohols (De Vuyst et al., 2014; Samoticha et al., 2019). Therefore, as ester precursors, esters formation leads to the decreasing of alcohols and acids. The esters formed by short‐chain acids are usually fruity, such as acetic acid ethyl ester and acetic acid hexyl ester (Li & Wang, 2013). Among them, ethyl ester with strong fruit and wine aroma is considered to play an important role in fermenting food and can impart its fruity aroma (De Vuyst et al., 2014). Consequently, the addition of *P. pentosaceu* as a leavening agent makes the fermentation aroma better and also makes Laomian steamed bread more popular with consumers. In the same vein, polymers produced from *P. pentosaceus* may be beneficially affect a number of technological properties of steamed bread, including water absorption of the dough, increased loaf volume, and retarded bread staling.

### 3.2 The population dynamic change of *S. cerevisiae* and *P. pentosaceus* during fermentation

**FIGURE 1:**
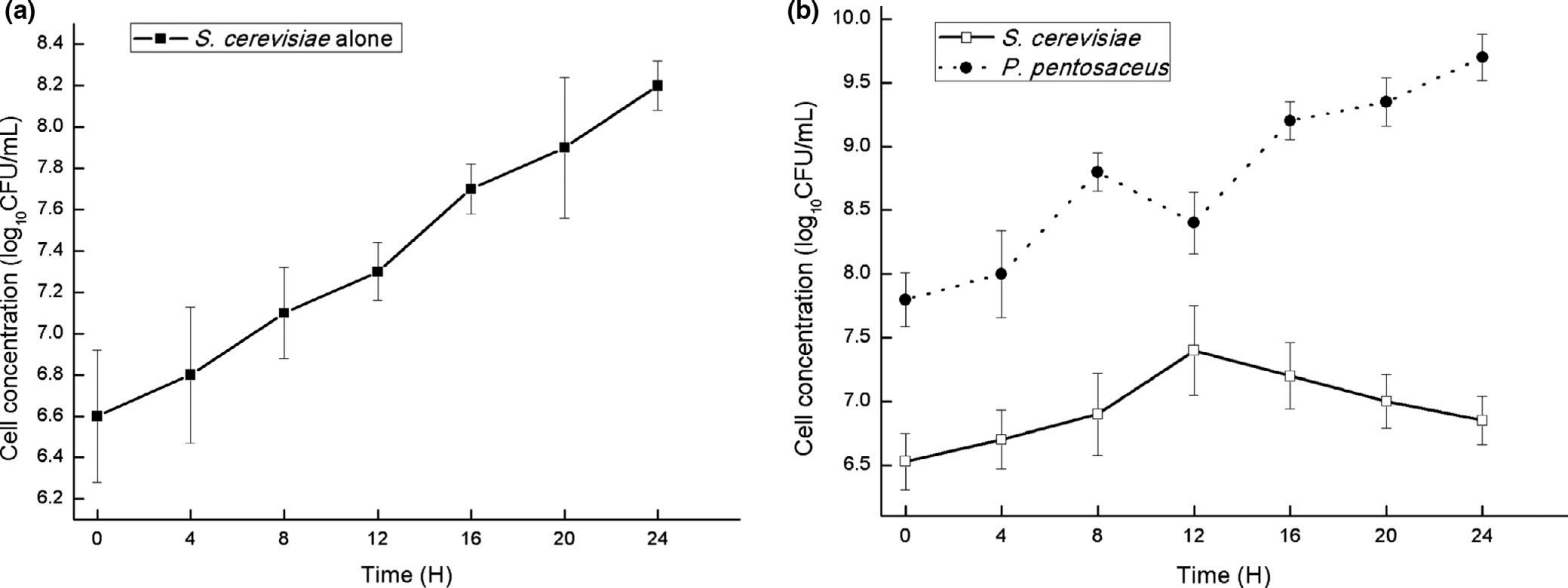
Growth changes of *S. cerevisiae* alone fermentation dough (A) and *S. cerevisiae* and *P. pentosaceus* joint fermentation dough (B) detected by RT‐qPCR

In order to gain insights into the kinetic interaction between *S. cerevisiae* and *P. pentosaceus* during the simulated fermentation, RT‐qPCR was used to monitor the cell quantities of *S. cerevisiae* and *P. pentosaceus*. For this propose, DNA was prepared from similar amounts of sourdough taken at different time points during fermentation. Amplification curves showed reproducible differences between the amount of strains special nucleic acid fragments, and the calculation results show that the correlation coefficients (*r^2^*) of the standard curves were 0.96 for *S. cerevisiae* and 0.97 for *P. pentosaceus*, which prove good correlations. Their growth trends were plotted. As showed in Figure 1a, when only *S. cerevisiae* was used as fermentation agent, the cell concentrations of *S. cerevisiae* continued to increase throughout 24 hr fermentation process. In the same vein, the cell concentrations of *S. cerevisiae* and *P. pentosaceus* joint fermentation increased steadily during the early stages of fermentation (Figure 1b). After 12 hr fermentation, the cell concentrations of *S. cerevisiae* reached the maximum, the cell concentrations of *P. pentosaceus* fluctuated lightly, and the reduction was 0.4 log_10_ CFU/mL. During the subsequent fermentation process, the cell concentrations of *S. cerevisiae* declined continuously, but the cell concentration of *P. pentosaceus* increased steadily and the final concentration increase was 1.9 log_10_ CFU/ml. Altogether, it is the first time to establish RT‐qPCR technology for real‐time monitoring the kinetics of *S. cerevisiae* and *P. pentosaceus* in the fermented dough. The establishment of this method greatly simplifies the detection procedure in Chinese traditional Laomian and improves the detection efficiency, and our results show that addition of *P. pentosaceus* did not result in the growth improvement of *S. cerevisiae*.

Mechanisms of *P. pentosaceus* in fermented products are complex. Relevant research shows that biochemical changes occur in the carbohydrate and protein components of the flour due to the action of microbial and flour indigenous enzymes during fermentation (Poutanen, Flander, & Katina, 2009). In the initial stage of simulated fermentation, the cell concentrations for *S. cerevisiae* and *P. pentosaceus* constantly increases due to the rich nutrition of wheat flour. *S. cerevisiae* consumes the sugars present in the flour at a higher rate than the *P. pentosaceus* (Cagno et al., 2014). When easy‐metabolizing sugars are exhausted, the cell concentration of *P. pentosaceus* fluctuates weakly while the cell concentration of *S. cerevisiae* reaches its maximum. Metabolic activity of *P. pentosaceus* causes acidity, *S. cerevisiae* is sensitive to nondissociated acetic acid, especially lactic acid (Minervini, Lattanzi, De Angelis, Di Cagno, & Gobbetti, 2012). These reasons led to a decrease in cell concentration of *S. cerevisiae* after 12 hr of fermentation. When *P. pentosaceus* is in low oxygen environment, the acid production efficiency is relatively low (Reijo et al., 2017), *S. cerevisiae* is to a certain extent tolerant toward a low pH and high osmotic pressure. Therefore, the cell concentration of *S. cerevisiae* slowly decreases in the later stage of fermentation, the cell concentration of *S. cerevisiae* still increases by 0.4 log_10_ CFU/ml compared with the start after 24 hr. Our results shows that jointly fermented dough contains metabolically active cells of *P. pentosaceus* at 10^8^–10^9^ CFU/g and *S. cerevisiae* at 10^6^–10^7^ CFU/g shows that a sponge (fermented dough) should, which is in line with previous research, mainly because *S. cerevisiae* can use invertase to hydrolyze sucrose into glucose and fructose (Vuyst, Vrancken, Ravyts, Rimaux, & Weckx, 2009), which serve as nutrients for the *P. pentosaceus*. Moreover, the hydrolysis releases monosaccharides about 200 times faster than fermentation (Araque, Bordons, & Reguant, 2012), and sucrose is quickly depleted during fermentation. Therefore, the cell concentration of *P. pentosaceus* displayed an upward trend in the later stage of simulated fermentation. In short, joint fermentation leads to the cell concentration of *S. cerevisiae* slight declining, and the cell concentration of *P. pentosaceus* continues to increase until 24 hr but a slight alteration at 12 hr. The two associations reflect the metabolic capabilities difference that is of importance in the ecosystem. With the growth of the *P. pentosaceus*, lactic and acetic acids formation result in the liberation of aliphatic compounds, dicarboxylic acids, and hydroxy amino acids, and proteases aid in the liberation of amino acids which explains why the joint fermentation produce more fermented aroma, and it is important consideration for consumer (Zhang, Zhang, Sadiq, Arbab, & He, 2019).

### 3.3 Extracellular protein composition *S. cerevisiae* and *P. pentosaceus*

**FIGURE 2:**
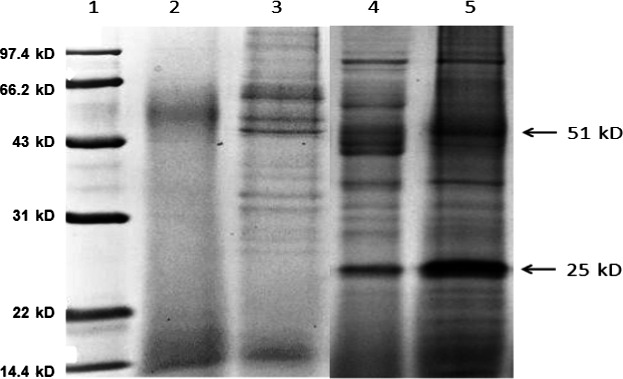
SDS‐PAGE photo for total extracellular proteins from *S. cerevisiae* alone or *S. cerevisiae* and *P. pentosaceus* joint fermentation

To investigate the aroma forming mechanism by *P. pentosaceus,* extracellular protease activity is a very important indicator. To detect extracellular proteins of *P. pentosaceus,* we extracted the whole extracellular proteins and used SDS‐PAGE to isolate the protein spectra, the protein gel electrophoresis results were shown in Figure 2. The molecular weight range of extracellular proteins extracted from *S. cerevisiae* is about 27k Da–100k Da, and there were 13 types of extracellular proteins with different molecular weights. The molecular weight range of extracellular proteins extracted from *P. pentosaceus* was about 25k Da–97k Da, and there were 12 types of extracellular proteins. There was no additional extracellular protein appearance for *S. cerevisiae* and *P. pentosaceus* joint extraction. To our interesting, the concentrations of 25 k Da and 51 k Da extracellular proteins from *P. pentosaceus* in joint culture increased significantly. In brief, our results display that there was no extra protein, joint culture resulted in the 25 k Da and 51 k Da proteins expression quantity increasing of *P. pentosaceus*.

*Pediococcus pentosaceus* usually has two important functions in the food field, one is to extent shelf life of food by producing antimicrobial peptides such as bacteriocin or pediocin and others (Kwon et al., 2018; Michalska et al., 2007), and another is to produce protease to improve flavors. Our study displays that *P. pentosaceus* can produce more extracellular proteins of 51.1k Da due to being induced by *S. cerevisiae,* which may be consistent with LysM peptidoglycan binding domain proteins based on the molecular weight, can inhibit the adhesion of pathogenic bacteria (Sun, Chen, & Wang, 2019). All these further indicated that traditional Laomian with *P. pentosaceus* had longer shelf life. And, the content of 25k Da extracellular protein also increases significantly. Latest paper shows that there were two kind of proteases, *P. pentosaceus* from Harbin dry sausages produced 29.6k Da of extracellular protease (Cardinali et al., 2018; Xu et al., 2010); *P. pentosaceus* isolated from fermented fish generated 37k Da of extracellular acidic protease (Yanshun et al., 2015). Therefore, we speculate that the 25 k Da of extracellular protein in this study may be same type of protease. It is certain that the secondary metabolites produced by *S. cerevisiae* caused *P. pentosaceus* to increase the 25k Da and 51.1k Da protein expression of or increase the transport efficiency, but mechanisms in details need to further study using proteomics.

### 3.4 Extracellular protease activity of *P. pentosaceus* in different pH conditions

**FIGURE 3:**
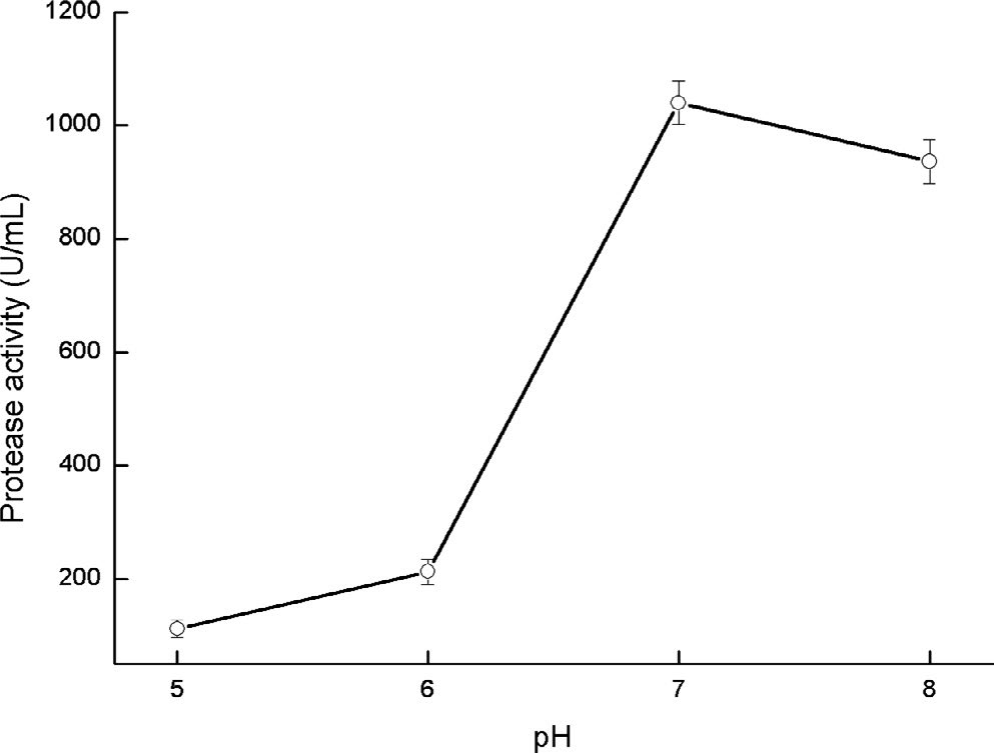
Extracellular protease activities of *P. pentosaceus* under different pH culture conditions

Dough fermentation often appeared acidity; in order to research extracellular protease activity of *P. pentosaceus* in different pH conditions, we used Folin–Ciocalteu method to detect extracellular protease activity. The result exhibits that correlation coefficients (*r^2^*) of the standard curve is 0.9994, which means an excellent linear relationship. Figure 3 exhibits the protease activities under different conditions (37°C, pH 5–8), and the maximum value is 1,039.71 U/ml at pH 7. And when the pH value is 8, the protease activity of *P. pentosaceus* decrease slightly, which is 936.06 U/ml. In contrast, when the pH value is 6, the protease activity decreased dramatically to 213.15 U/ml. As the pH value is 5, the extracellular protease activity of *P. pentosaceus* continues to decrease, reaching a minimum value of 112.49 U/ml. Conclusively, under neutral and weak alkaline culture conditions, the extracellular protease activity of *P. pentosaceus* is higher, while acidic culture conditions are not conducive to the extracellular protease activity of *P. pentosaceus*.

Traditional Laomian is a kind of fermentation agent naturally selected by environmental microorganisms, mixed with dough in the amount of 10%–30%, generally. The selection of sourdough microbiota occurs spontaneously during daily refreshment (also called backslopping) (Taccari et al., 2016). Therefore, the whole system is weakly alkaline in the initial stage of traditional Laomian fermentation. Our research results show that the activity of *P. pentosaceus* extracellular protease was higher in both neutral and weakly alkaline environments, which also reveals the reason for its increasing cell concentration in the early stages of fermentation. As the fermentation time increases, *P. pentosaceus* constantly produces acid, and the accumulation of organic acids causes the overall pH to decrease continuously. As the pH value decreases, protease activity continues to decrease, especially at pH 6. The occurrence of *P. pentosaceus* reflects its adaptability to the prevailing Laomian fermentation conditions. *Pediococcus pentosaceus* extracellular proteases play a role in breaking down the macromolecules proteins into amino acids which is also precursor of esters (Li & Wang, 2013), so that the fermentation dough with *S. cerevisiae* and *P. pentosaceus* has richer volatile aroma substances. China is a great country of bread consumption(Huang, 2014), to our knowledge, it is the first time to study the aroma substances associated with mechanism of *P. pentosaceus* in fermentation dough by researching dynamic changes, extracellular proteins and extracellular protease activity, and our research will benefit to explore the fermentation characteristics of *P. pentosaceus* from Chinese traditional Laomian and develop some new type joint fermentation starter.

## 4 CONCLUSION

*Pediococcus pentosaceus* is important probiotics and widely exists in Chinese Laomian. Simulated fermentation shows that addition of *P. pentosaceus* results in significant increase in the type and content of esters in fermented dough. Additional eight kinds of esters were produced, and among them, the concentrations of acetic acid hexyl ester, formic acid octyl ester, and heptanoic acid ethyl ester rise drastically. Especially, the most dramatic changes in content are hexanoic acid ethyl ester, and the concentration increases from 9.12% to 29.73%. Due to nutritional competition and microbial growth habits, the cell concentration of *S. cerevisiae* decreases slightly by 0.4 log_10_ CFU/ml; the cell concentration of *P. pentosaceus* increases by 1.9 log_10_ CFU/ml in the end of the simulated fermentation, the metabolic activity of *S. cerevisiae* provided nutrients for the growth of *P. pentosaceus*. With the addition of *S. cerevisiae*, the 25 k Da and 51 k Da extracellular proteins quantities of *P. pentosaceus* increase significantly. The extracellular protease activity of *P. pentosaceus* was best at pH 7, which was 1,039.71 U/ml. As the pH value decreases, the extracellular protease activity continues to decrease. However, the decrease rate is fast. In conclusion, with the addition of *S. cerevisiae*, the expression of *P. pentosaceus* extracellular proteases increases during the fermentation process, resulting in a dramatic increase in the volatile aroma substances. As the fermentation continues, the cell concentration of *S. cerevisiae* decreases slightly, and the secondary metabolites of *S. cerevisiae* provide nutrients for the growth of *P. pentosaceus*. This research systematically explores the fermentation characteristics of *P. pentosaceus* in Chinese traditional Laomian and benefits the development of Chinese traditional Laomian bread.

## AUTHOR CONTRIBUTIONS

Yunxia Gong involved on experimental research and writing—original draft preparation. Xiaobao Qi involved in design, formal analysis and writing—review and editing.

## ETHICAL STATEMENT

The authors declare that they had no competing interests. The authors declare that there is no study involving human/animals subjects.

## Supporting information

Fig S1
