# Supplementary material for: A study revealing volatile aroma produced by Pediococcus pentosaceus in dough fermentation
Source: Food Sci Nutr. 2020 Aug 3;8(9):5077–85. doi: 10.1002/fsn3.1807 (PMC7500783; doi:10.1002/fsn3.1807)
Supplement: Supplementary file 1 — Fig S1 [file FSN3-8-5077-s001.docx]

**Supplementary Materials:** The following are available online at www.mdpi.com/xxx/s1, Figure S1: Chromatograph peaks of major volatile compounds of fermentation doughs by *S. cerevisiae* alone (A) or *S. cerevisiae* and *P. pentosaceus* joint (B).


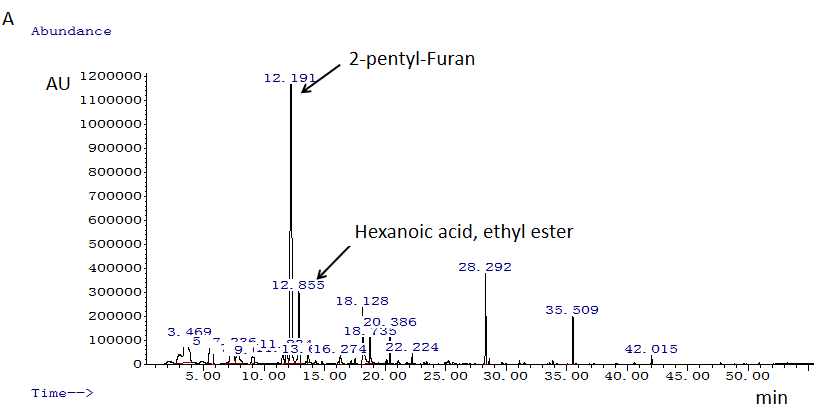

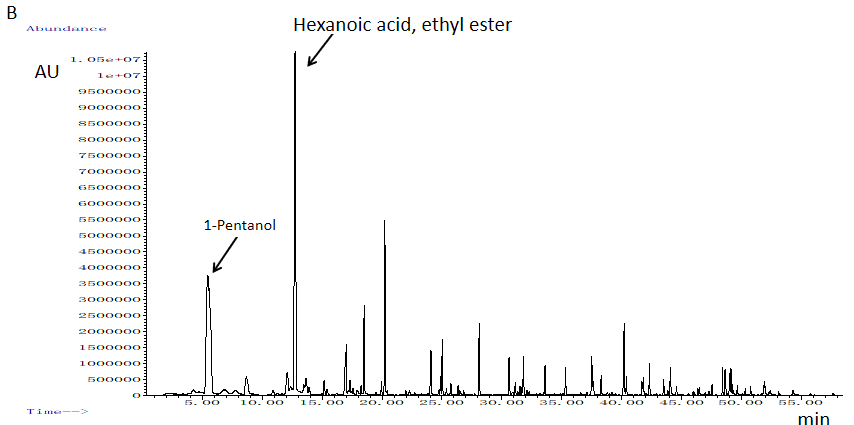


Figure S1. Chromatograph peaks of major volatile compounds of fermentation doughs by *S. cerevisiae* alone (A) or *S. cerevisiae* and *P. pentosaceus* joint (B). Fig.S1A shows that volatile aroma substances produced by *S. cerevisiae* fermentation dough, extremely high levels of 2-pentylfuran, a fruit-aroma substance, were detected. Fig.S1B shows that volatile aroma substances produced by *S. cerevisiae* and *P. pentosaceus* fermentation dough, the content of hexanoic acid ethyl esters is extremely large (29.7%), and the content of 1-pentanol increases obviously.
